# Dietary fiber modulates gut microbiome and metabolome in a host sex-specific manner in a murine model of aging

**DOI:** 10.3389/fmolb.2023.1182643

**Published:** 2023-06-15

**Authors:** Saurabh Kadyan, Gwoncheol Park, Bo Wang, Ravinder Nagpal

**Affiliations:** ^1^ Department of Nutrition and Integrative Physiology, College of Health and Human Sciences, Florida State University, Tallahassee, FL, United States; ^2^ Department of Biomedical and Chemical Engineering and Sciences, Florida Institute of Technology, Melbourne, FL, United States

**Keywords:** prebiotics, inulin, fiber, gut dysbiosis, metabolites, metabolomics, microbiota, sexual dimorphism

## Abstract

Emerging evidence reveals the fundamental role of the gut microbiome in human health. Among various factors regulating our gut microbiome, diet is one of the most indispensable and prominent one. Inulin is one of the most widely-studied dietary fiber for its beneficial prebiotic effects by positively modulating the gut microbiome and microbial metabolites. Recent research underscores sexual dimorphism and sex-specific disparities in microbiome and also diet-microbiome interactions. However, whether and how the prebiotic effects of dietary fiber differ among sexes remain underexplored. To this end, we herein examine sex-specific differences in the prebiotic effects of inulin on gut microbiome and metabolome in a humanized murine model of aging i.e., aged mice carrying human fecal microbiota. The findings demonstrate that inulin exerts prebiotic effects, but in a sex-dependent manner. Overall, inulin increases the proportion of *Bacteroides*, *Blautia,* and glycine, while decreasing *Eggerthella*, *Lactococcus*, *Streptococcus*, trimethylamine, 3-hydroxyisobutyrate, leucine and methionine in both sexes. However, we note sex-specific effects of inulin including suppression of *f*_*Enteroccaceae:_*, *Odoribacter*, bile acids, malonate, thymine, valine, acetoin, and ethanol while promotion of *Dubosiella*, pyruvate, and glycine in males. Whereas, suppression of *Faecalibaculum, Lachnoclostridium, Schaedlerella,* phenylalanine and enhancement of *Parasutterella, Phocaeicola, f_Lachnospiraceae;_, Barnesiella, Butyricimonas*, glycine, propionate, acetate and glutamate are observed in females. Altogether, the study reveals that prebiotic mechanisms of dietary fiber vary in a sex-dependent manner, underscoring the importance of including both sexes in preclinical/clinical studies to comprehend the mechanisms and functional aspects of dietary interventions for effective extrapolation and translation in precision nutrition milieus.

## 1 Introduction

Emerging evidence has highlighted the profound impact of the gut microbiome on our physiology and health (Jovel et al., 2018). Our gut is densely packed with a highly diverse and complex community of microorganisms, which via their specific metabolic activities continuously interact with the intestinal epithelium leading to distinct physiological, metabolic, and immunomodulatory responses either locally or systemically within the host (Nagpal et al., 2016). The diversity and metabolic capacity of the gut microbiome are strongly and tightly regulated by the dietary lifestyle of the host throughout the lifecycle. Moreover, the evolution of gut microbiome with the aging process is closely linked to dietary patterns with a fiber-deficit diet being often correlated with reduced gut bacterial diversity and increased propensity to various intestinal, cardiometabolic, and neurobehavioral disorders (Nagpal et al., 2018a; Ghosh et al., 2022). Recent studies by us and others have also highlighted the existence of considerable sexual dimorphism in the gut microbiome, indicating that dietary interventions may influence gut microbiota in a sex-dependent manner (Bolnick et al., 2014; Suzuki et al., 2017). Despite the significant influence of sex-diet interactions on the gut microbiome, most clinical studies examining the effects of dietary fiber intake have overlooked the phenomenon of sexual dimorphism in the gut microbiome (Dominianni et al., 2015; Chu et al., 2019; Reimer et al., 2020; Vijay et al., 2021), with few noted exceptions (Trimigno et al., 2017; Reider et al., 2020; Benítez‐Páez et al., 2021). Besides, dietary studies involving preclinical models mostly use only males to avoid confounding effects of the estrus cycle in females and thus fail to examine sex-based differences (Shastri et al., 2015). Since the precise mechanisms underlying the relationship between diet, sex and microbiome/metabolome are only beginning to be understood, it becomes imperative to investigate the mechanisms of dietary components including fibers and prebiotics to validate the universality of their health effects while avoiding the extrapolation of the prebiotic effects of males on females or *vice versa*.

Of all the dietary fibers, inulin is the most extensively studied one for its prebiotic properties and beneficial modulation of the gut microbiome by promoting short-chain fatty acids (SCFAs) in the distal gut leading to enhanced intestinal homeostasis, immune-stimulation, blood glucose, and lipid regulation (Tawfick et al., 2022). Inulin is composed of 2–60 linear fructose units joined together via β-(1 → 2)-D-fructosyl-fructose glycosidic bonds along with a glucose unit at the terminal end attached via α-(1 → 2) linkage. Despite the increasing recognition of host sex as a major confounder in microbiome studies, the mechanistic preclinical studies exploring the health effects of inulin are largely confined to male rodents (Zhang et al., 2017; Du et al., 2020; Li et al., 2020; Shao et al., 2020; Régnier et al., 2023). Animal models are widely used for understanding the mechanistic and functional basis of health benefits imparted by dietary fibers but suffer from limitations of differences in their gut microbial community structure compared to humans (Li and Zhang, 2022). For instance, the relative abundance of 79 genera found in mouse and human gut varies greatly in their relative abundance patterns (Nguyen et al., 2015; Nagpal et al., 2018b). Animal models colonized with human microbiota offer an attractive avenue to address this problem and may provide close reflections of dietary impacts which are ecologically similar to the human gut (Lundberg, 2019). However, there is a lack of evidence elucidating the prebiotic effects of inulin on gut microbiome-metabolome arrays in humanized murine models of aging, particularly taking into consideration the indirect influence of a sexually dimorphic gut. Keeping these knowledge gaps in view, we herein aim to determine the extent to which inulin supplementation within a western-style diet ameliorates gut microbiome and metabolome pool towards a healthy ecosystem in a humanized mouse model of aging and whether and how these prebiotic effects differ according to host sex. The study reveals that inulin modulates gut microbiome and its metabolites in a sex-dependent manner, thereby signifying the prevalence of sexual dimorphism in the gut microbiome which may differentially regulate the diet-microbiome interface as well as the therapeutic attributes delivered by dietary fiber.

## 2 Material and methods

### 2.1 Study design

Animal experiments were executed as per our previously described protocol (Kadyan et al., 2023). Briefly, old male and female C57BL/6J mice (55 weeks old) were subjected to a gut-depletion procedure using the antibiotic cocktail (ampicillin [1 g], Metronidazole [1 g], Neomycin [1 g], and Vancomycin [0.5 g] per liter drinking water) regimen for 4 days followed by polyethylene glycol treatment (200 μL per dose per mouse; 425 g/L) to cleanse remnant gut microbiota. Pooled fecal samples from five healthy older adult human donors (3M, 2F; 50–55 years old) were transplanted (via oral gavage) in mice after followed by their random allocation into two experimental groups (N = six to eight per group per sex): CTL (western-style high-fat diet control) and INU (high-fat diet supplemented with inulin (Biosynth) @ 5% w/w). The dietary regimen continued for 20 weeks. The fecal samples for metabolome and microbiome analysis were collected and stored at −80°C till further analysis. The study was carried out in accordance with the recommendations and guidelines of the Institutional Animal Care and Use Committee. The protocol was approved by the Institutional Animal Care and Use Committee at Florida State University (PROTO202100008).

### 2.2 Gut microbiome analysis

The gut microbiome was measured as per our previously described methods (Nagpal et al., 2018a; Ahmadi et al., 2019; Nagpal et al., 2019; Nagpal et al., 2021; Saccon et al., 2021; Clark et al., 2022; Munley et al., 2022). Briefly, genomic DNA from 200 mg of the fecal specimen was extracted using the QIAmp PowerFecal Pro DNA Kit (Qiagen) according to the manufacturer’s instructions. The amplification of the hypervariable V4 region of bacterial 16S rRNA gene using Universal primers 515F (barcoded) and 806R was done in accordance with the Earth Microbiome Project benchmark protocol (https://earthmicrobiome.org/). Equal molar concentrations of the library were pooled and sequenced for paired-end (2 × 300 bp) sequencing using an Illumina MiSeq sequencer (using Miseq reagent kit v3; Illumina Inc., San Diego, USA). QIIME2 (ver. 2–2022.11) was used for microbiome bioinformatics analysis (Bolyen et al., 2019). The raw sequences were quality-filtered, trimmed, and denoised through DADA2 pipeline (Callahan et al., 2016). All identified amplicon sequence variants (ASVs) were aligned with the MAFFT (Katoh et al., 2002). ASVs were assigned with a naïve Bayes taxonomy classifier developed for the sklearn classifier against the pre-built from the most recent version of Greengenes2 database (ver. 2022.10) (Bokulich et al., 2018; McDonald et al., 2022).

### 2.3 Global untargeted metabolomics analysis

The collected fecal samples were processed as per the previously described protocol (Gratton et al., 2016) with slight modifications. Briefly, samples were extracted using deionized water by vertexing for 5 min. The extracted samples were mixed with a phosphate buffer (pH = 7.4) in D_2_O which made the final solution with 10% D2O, 0.1 M phosphate, and 0.1 mM Trimethylsilyl propionate (TSP). The samples were centrifuged and transferred to 5 mm NMR tubes for further data acquisition by a Bruker Ascend 400 MHz high-resolution NMR (Bruker Biospin, Germany. The 1D first increment of a NOESY (noesygppr1d) experiment with water suppression was applied for all samples with 64 scans. All NMR spectra were phased and referenced to TSP in TopSpin 4.06 (Bruker BioSpin, Germany). All the NMR processing will be carried out in Amix 4.0 (Bruker BioSpin) and the NMR spectra will be bucketed using our previously reported automatic method (Wang et al., 2020) to minimize peak overlap and splitting. Metabolite indentation will be carried out using Chenomx 8.6 (Chenomx Inc.). Total intensity normalization was applied before further data analysis.

### 2.4 Bioinformatics and statistical analysis

‘R’ or ‘Python’ packages were used for downstream microbiome and metabolome analyses and visualization. Observed features (ASVs), Faith PD (phylogenetic diversity), and Shannon (species richness and evenness) index were used as alpha-diversity metrics, and weighted UniFrac distance and Bray-Curtis dissimilarity index were used for the analysis of beta-diversity in the form of the principal coordinate analysis (PCoA). To identify differentially abundant taxa and/or metabolites between CTL and INU groups, the linear discriminant analysis (LDA) effect size (LefSe) (Segata et al., 2011), ANOVA-Like differential expression (ALDEx2) method (Fernandes et al., 2014), and multivariate analysis by linear models (MaAsLin2) (Mallick et al., 2021) were used. *p*-values were corrected using the Benjamini–Hochberg procedure. Cladograms were generated using differential features with LDA>2.0. For the prediction of microbiome and metabolites via supervised classification, a q2-sample-classifier plugin for the QIIME2 via nested stratified 4-fold cross-validation with Random Forest classifier grown with 2,000 trees was used. Spearman’s correlation was used to identify significant correlations between groups.

## 3 Results

### 3.1 Inulin modulates gut microbiome diversity and metabolomic profiles in a sex-dependent manner

**FIGURE 1:**
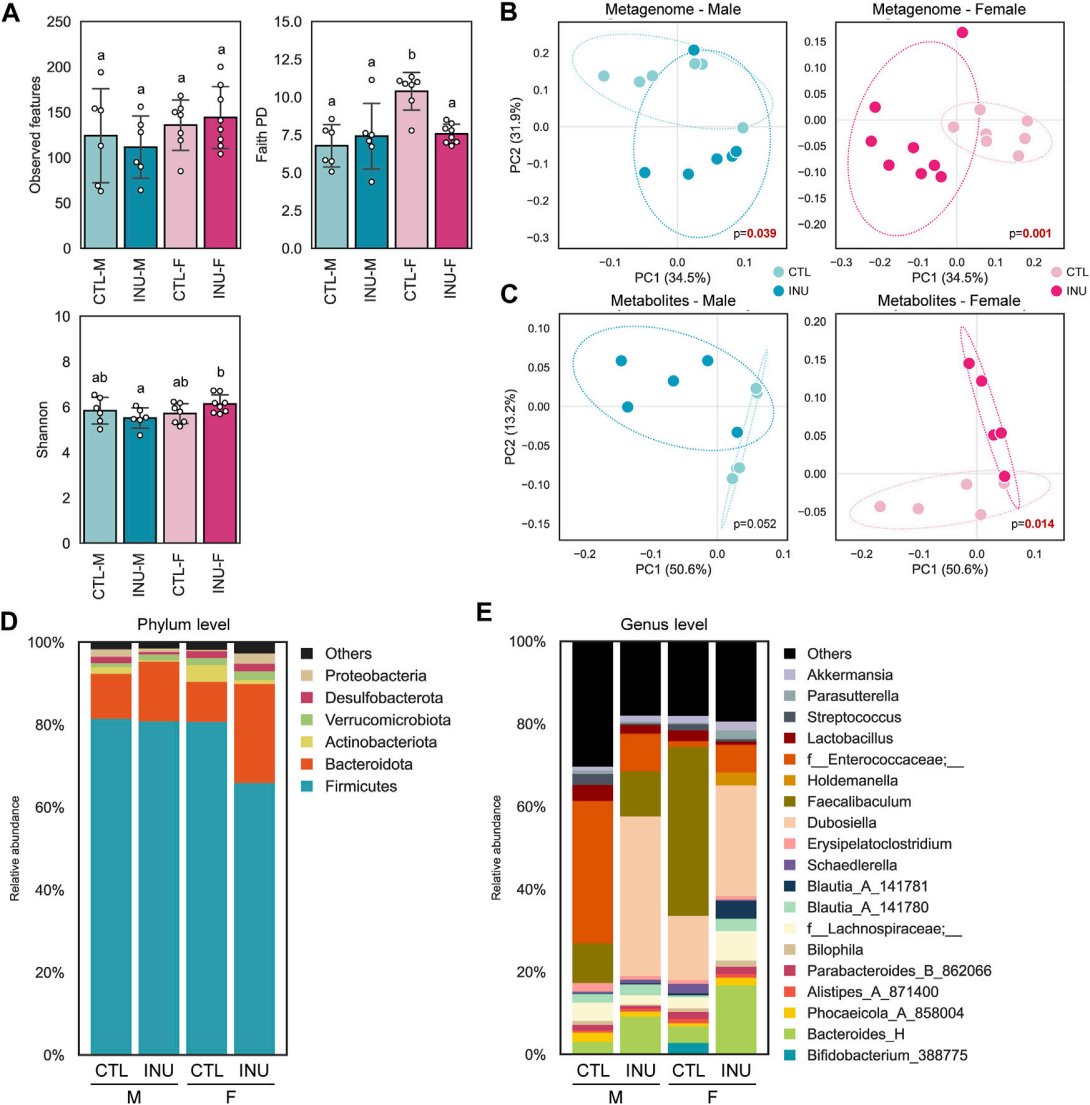
Sex-specific effects of inulin on gut microbiome diversity, fecal metabolites, and taxonomic hierarchies. **(A)** Microbial diversity of each group determined using the number of observed features, Faith PD, and Shannon index with differences between groups assessed using the pairwise Kruskal–Wallis test and different letters indicate significant differences (*p* < 0.05). PCoA plots of **(B)** weighted UniFrac distances between samples based on ASVs and **(C)** Bray-Curtis dissimilarity between samples based on metabolites composition. Bacterial composition at **(D)** phylum and **(E)** genus level.

The intra-individual diversity (⍺-diversity; microbial species richness) in males and females is evaluated using observed features, Faith PD, and Shannon indices (Figure 1A). No significant differences are observed in the INU group of both sexes; however, Faith PD scores are significantly higher in females than males and are subsequently decreased after INU treatment. The inter-individual diversity (β-diversity), assessed using PCoA based on weighted UniFrac distances, shows significantly distinct arrays between CON vs. INU groups in both sexes (Figures 1B, C). However, fecal metabolome arrays, as assessed via PCoA of the Bray-Curtis dissimilarity index, exhibit relatively more distinct signatures in females than males after INU treatment (Figure 1D). INU treatment shows marked differences (*p* < 0.014) in the metabolite profiles in females while a similar differential trend (*p* < 0.052) is also observed in males relative to their CTL groups.

Sex-specific differences in the microbiome modulation by INU treatment are further observed using altered bacterial abundance at bacterial phylum (Figure 1E) and genus level (Figure 1F). At the phylum level, nearly 90% of relative abundance is contributed by Firmicutes and Bacteroidota for all groups. Amongst all, the INU-F group differs from others by exhibiting enhanced and reduced abundance of phyla *Bacteroidota* and Firmicutes, respectively. Further analysis of genus level reveals interesting patterns of bacterial abundance in both sexes. In males, relative abundance of genera *Bacteroides_H* and *Dubosiella* are increased, whereas *f_Enterococcaceae;_*, *f-Lachnospiracea*e; _, *Erysipelatoclostridium* and *Streptococcus* are decreased after INU intervention. In contrast, INU treatment in females promotes the prevalence of *Bacteroides_H, f-Lachnospiracea*e*;_, Blautia_A*, *Dubosiella*, *f_Enterococcaceae;_* and *Parasutterella* while that of *Bifidobacterium_388775, Schaederella,* and *Faecalibaculum* are reduced compared to CTL-F. Altogether, these findings demonstrate that inulin modulates the gut microbiome and metabolome in a sex-dependent manner.

### 3.2 Inulin exerts sex-specific modulation in gut microbiome-metabolome arrays and metabolic pathways

**FIGURE 2:**
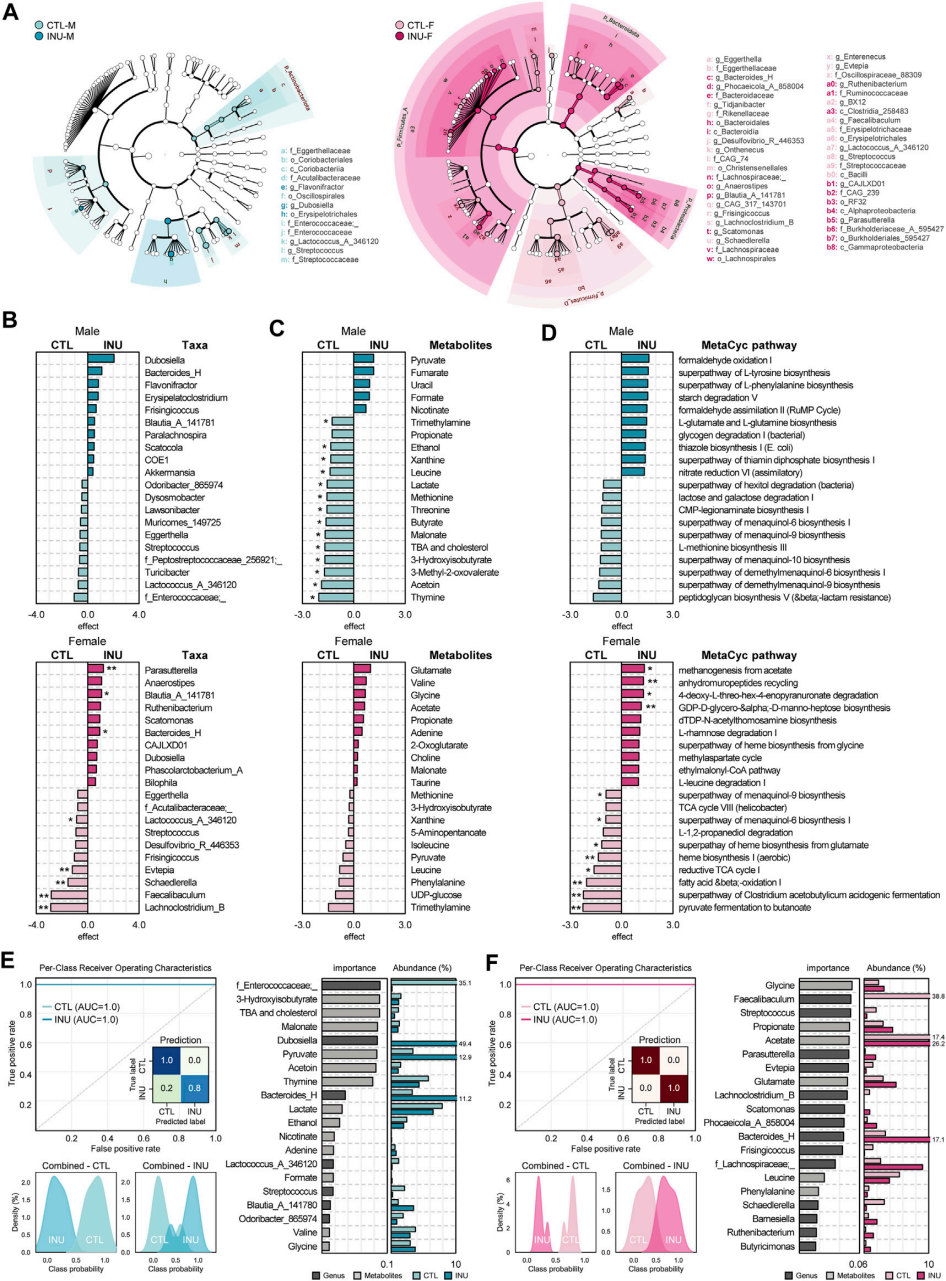
Sex-dependent disparities in the effects of inulin on unique features of microbiome, metabolome, and metabolic pathways. **(A)** Bacterial composition differences between groups were detected using LEfSe analysis (LDA >3.0) and depicted in a cladogram. Top 10 **(B)** taxa, **(C)** metabolites, and **(D)** MetaCyc pathways for each sex determined using ALDEx2 (* and ** indicate q < 0.1, and q < 0.05 respectively). Random forest prediction between groups in each **(E)** male and **(F)** female using the combined abundance data of bacterial genera and metabolites. Receiver Operating Characteristic (ROC) curve and prediction rates heatmap represents the classification accuracy, and probability histograms, respectively along with top 20 most strongly predictive genera and metabolites by relative importance score and their average abundance (%) for each group.

Unique gut microbiome signatures induced by inulin supplementation in males and females are assessed using two benchmark statistical approaches: LEfSe and ALDEx2. The taxonomic hierarchy of differentially abundant features, as analyzed using LEfSe cladograms (Figure 2A) clearly demonstrate that INU treatment exerts a more profound effect in females than males. Subsequent execution of the more conservative and precise statistical algorithm ALDEx2 for differential abundance assessment of effect size (Nearing et al., 2022) depicts several key taxa that are increased or decreased by INU relative to control (Figure 2B). While no significant differences in the abundance of key taxa are seen in males, females show a significant promotion in the abundance of *Parasutterella* (q < 0.05)*, Blautia_A_141781* (q < 0.1) and *Bacteroides_H* (q < 0.1) in addition to significantly (q < 0.05) decreased abundance of *Evtepia, Schaedlerella, Faecalibaculum,* and *Lachnoclostridium_B*. Likewise, key metabolites modulated by INU treatment also vary among sexes (Figure 2C). Considerable variation (q < 0.1) in metabolites abundance pattern is exhibited only in males, which includes a reduction in metabolites including trimethylamine, ethanol, thymine, acetoin, butyrate, 3-hydroxyisobutyrate, 3-methyl-2-oxovalerate, malonate, threonine, methionine, lactate, leucine, xanthine, total bile acids (TBA) and cholesterol. Though insignificant, trimethylamine also follows a reducing trend in females. Subsequently, to predict and assess modulations in the metabolic processes originating from differences in the gut microbiome in both sexes, we execute and analyze experimentally elucidated MetaCyc pathway database. As summarized in Figure 2D, overall, in line with metabolomics data, the MeTaCyc functional pathways also modulated differently in males and females; however, only females show significantly (q < 0.05) affected pathways including upregulation of anhydromuropeptides recycling and GDP-D-glycero-α-D-manno-heptose biosynthesis together with downregulation of heme biosynthesis I (aerobic), fatty acid β-oxidation, superpathway of *Clostridium acetobutylicum* acidogenic fermentation and pyruvate fermentation to butanoate.

We subsequently also execute machine-learning algorithm based on random-forest prediction model of integrated microbiome and metabolite profiles, which clearly predicts differences in CTL vs. INU groups among both sexes. The top 20 strongly predictive genera and metabolites each for males and females are presented in Figures 2E, F. Among males, *f_Enterococcaceae;_*, *Streptococcus*, *Odoribacter_865974*, *Lactococcus_A_346120*, 3-hydroxyisobutyrate, thymine, acetoin, lactate, ethanol, valine, TBA and cholesterol are more predictive of CTL group, whereas, *Dubosiella, Bacteroides_H, Blautia_A_141780*, pyruvate, adenine, and glycine are suggestive for INU group. Similarly, for females, *Faecalibaculum, Streptococcus, Evtepia, Lachnoclostridium_B, Frisingicoccus, Schaedlerella*, leucine, and phenylalanine predict CTL while *Parasutterella, Scatomonas, Bacteroides_H, f_Lachnospiraceae;_, Barnesiella, Ruthenibacterium, Butyricimonas*, glycine, propionate, acetate, and glutamate predict post INU intervention. Overall, these data infer that inulin modulates the gut microbiome structure and its functional metabolic pathways more prominently in females while its prebiotic effects on fecal metabolites are more marked in males.

### 3.3 Integrated co-regulation networks reveal sex-specific effects of inulin on microbiome, metabolome, and physiological responses

**FIGURE 3:**
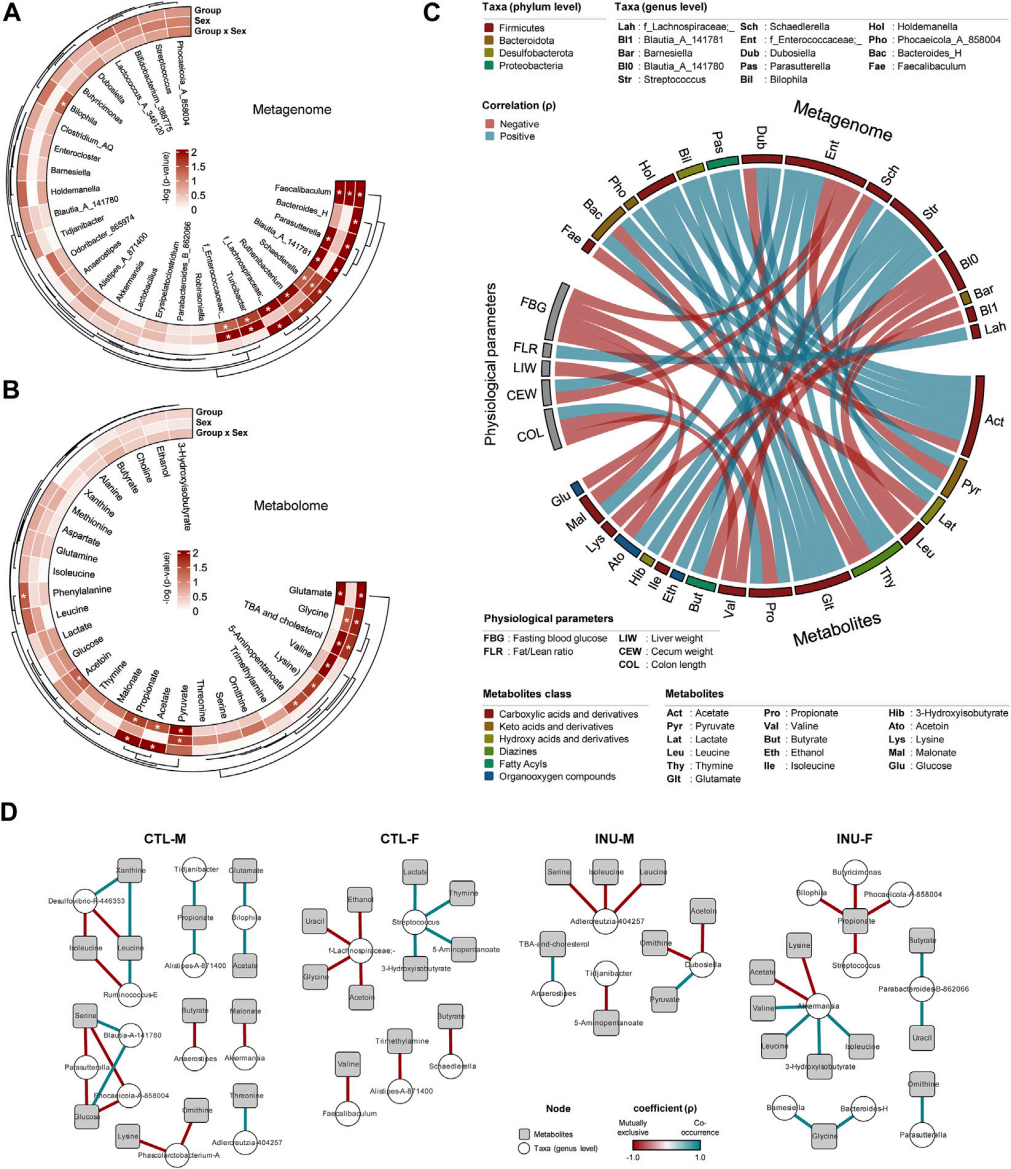
Inulin differently modulates gut microbiome-metabolome interactions together with physiological responses in male versus female mice. Circular heatmap shows differences of dominant **(A)** bacterial genera and **(B)** metabolites by treatment group and sex determined using two-way ANOVA models. **(C)** Correlation between most abundant 20 genera, 20 metabolites, and physiological parameters (Spearman’s rank correlation coefficient (ρ) > 0.55). **(D)** Bacterial genera-metabolites correlation network of each group. Each node represents one genus (white circle) and metabolites (gray rectangle), and only significant links are shown (Spearman’s rank correlation coefficient (ρ) > 1.0, Benjamini–Hochberg corrected *p*-value <0.05).

The variations in the metagenome (Figure 3A) and metabolome (Figure 3B) post-inulin intervention is evaluated using 2-way ANOVA using Group (CTL vs. INU) and Sex (males vs. females) as two independent factors as well using the interaction effects among these two factors (Group x Sex). *Bacteroides_H* is the only taxa significantly influenced by group alone. In addition to the group, the interaction effects show modulation in the abundance of *Parasutterella*, *Blautia_A*, *Ruthenibacterium,* and f*_Lachnospiraceae*. *Turcibacter* and *f_Enterococcaceae* are only affected by sex and group-sex interaction effects, while *Bilophila* is marginally influenced by interaction effects. The abundance of *Faecalibaculum* and *Schaedlerella* is affected by both factors and their interaction. From a metabolome viewpoint, the abundance of phenylalanine is solely explained by group, while glutamate, acetate, and propionate are influenced by group and interaction effects. The variation in glycine, TBA, and cholesterol is governed by group and sex, while the abundance of valine, lysine, and 5-aminopentanoate is only explained by sex.

The Spearman correlation network analyses reveal the intricate relationship between gut metagenome, metabolome, and host physiological responses associated with inulin intervention (Figure 3C). The details of physiological parameters evaluated for the current study are presented in Supplementary Figure S1. Intestinal tissue measures i.e., total intestine length, colon length, cecum weight, and liver weight were adjusted by normalization with bodyweight. The data show that fasting blood glucose is negatively correlated with *f_Enterococcaceae;_*, lactate, malonate, and acetoin and is significantly higher (*p* < 0.048) for the INU-M group (Supplementary Figure S1). Fat/lean mass ratio correlates positively only with f*_Lachnospiraceae;_.* Liver weight is negatively correlated with propionate. Cecum weight, which is consistently higher for both sexes of the INU group (Supplementary Figure S1), is positively and negatively associated with *Dubosiella* and *f_Enterococcaceae;_,* respectively. Colon length is positively associated with propionate but negatively with valine and glucose. Acetate and glutamate are directly associated with *Bacteroides_H, Phocaeicola_A, Bilophila,* and *Holdemanella*, the latter being also positively linked to propionate. Lactate is positively associated with *Streptococcus* only. The abundance of *Blautia_A_141780* is negatively linked to leucine, butyrate, and isoleucine. *Blautia_A_141781* and *Faecalibaculum* inversely correlate with lysine and acetate, respectively.

Further insights into microbiome-metabolome interplay are assessed using cooccurrence networks within each group (Figure 3D). In the CTL-M group, *Desulfovibrio-R-446353* exhibits a positive association with xanthine and an inverse association with leucine and isoleucine. The presence of serine and glucose directly correlates with *Blautia-A-141780* but inversely with *Parasutterella* and *Phocoaeicola-A-858004*. In the CTL-F group, the abundance of *f-Lachnospiraceae;_* is mutually exclusive for the presence of ethanol, uracil, glycine, and acetoin, while the abundance of *Streptococcus* co-occurs with the presence of lactate, thymine, 5-aminopentanoate, and 3-hydroxyisobutyrate. INU treatment marks a change of association between microbiome and metabolites for both sexes. *Anaerostipes,* which was negatively associated with butyrate in the CTL-M group, is positively associated with TBA and cholesterol in the INU-M group. Besides, serine, isoleucine, and leucine show a negative association with *Adlercreutzia-404257* post-INU treatment. Correlation network analyses for INU-F mainly center around *Akkermansia* and propionate. *Akkermansia* is positively associated with valine, leucine, 3-hydroisobutyrate, and isoleucine while negatively with acetate and lysine. Propionate abundance is inversely associated with *Bilophila, Butyricimonas, Streptococcus,* and *Phocaeicola A-858094*. Altogether, inulin significantly modulates the gut bacterial architecture and associated pool of metabolites but with distinct arrays of microbiome-metabolome cooccurrence in males *versus* females.

## 4 Discussion

Rodent models are invariably used to validate as well as understand the mechanisms and health effects of functional foods including dietary fibers. Given the increasing awareness of the sex-based differences in microbiome, metabolism and immunity, the validation of dietary fibers merits evaluation in rodents of both sexes to establish the universality of prebiotic effects. Accordingly, we herein apply ecosystem-wide gut microbiome and metabolome profiling to assess the prebiotic attributes of inulin fiber in modulating the microbiome-metabolome arrays in western-style diet-fed C57BL/6 aged mice carrying human gut microbiota wherein we find that these effects vary between males and females, further emphasizing the consideration and inclusion of sex-effects for extrapolation of health effects of dietary fibers.

Age, along with sex, is a confounding factor in gut microbiome studies due to the transition of the intestinal microbial community towards ‘dysbiosis’ as individuals age. While inulin had demonstrated its responsiveness in elderly population (55–80 years) by promoting abundance of beneficial taxa like *Bifidobacterium, Parabacteroides* and *Anaerostipes* together with reductions in fecal isobutyric acid levels (Kiewiet et al., 2021), emerging studies have revealed its age-dependent response in modulating gut microbiome and SCFAs composition. Recent preclinical studies have demonstrated diminished potential of inulin-induced microbiome shifts with age, which includes less diverse microbiomes compared to adult mice, age-dependent decline of fecal propionate acids and no significant effects on systemic inflammatory and gut health markers (Muthyala et al., 2022; Hutchinson et al., 2023). Nevertheless, future studies can overcome these deficits in the efficacy of inulin by investigating the dose-dependency of inulin supplementation and/or incorporating dietary fibers earlier in life, before reaching old age. In our current investigation, we further emphasize that sex is an important contributing factor that should be taken into account when targeting precision nutrition in old age.

Overall, inulin has insignificant effect on the combined estimators of microbial diversity and richness in either sex. A previous study also indicated no such effect on fecal community diversity in both sexes of rats fed with oligofructans supplemented standard chow diet (Shastri et al., 2015). However, another study demonstrated higher Shannon diversity in female gnotobiotic mice than males after fecal transplantation with male human donors, which also partly aligns with our study wherein CTL-F show high phylogenetic diversity than CTL-M even though there are no visible differences in Shannon diversity (Wang et al., 2020). This further posits that ecologically distinct environments between males and females eventually influence their gut microbiomes. For instance, the driving effect of sex in shaping the microbiota colonization pattern in gnobiotic mice after fecal transplantation from human donor on inulin-enriched diet had been acknowledged before (Wang et al., 2016). Although the precise mechanisms underlying sexual dimorphism in microbiome remain unclear, sex-specific differences in immune responses and hormones, and their direct or indirect effects on gut microbes have begun to unravel these phenomena (Bolnick et al., 2014). Sex hormones can interact with the gut microbiome in a bidirectional manner (Yoon and Kim, 2021). Firstly, the deconjugation of estrogens and androgens by gut microbiome alters their abundance profile in the intestine and systemic circulation. Secondly, estrogens and androgens demonstrate varying effects on the immune response, which in turn impacts the composition of the gut microbiota and intestinal permeability differently in males and females. High levels of estradiol in females and testosterone in males were associated with diverse gut microbiome (Shin et al., 2019). Thus, it is reasonable to assume that the alteration in microbial colonization patterns mediated by host sex hormones and immune system also modulate diet-microbe interactions in the gut ecosystem in response to intake of dietary fibers.

Sex-specific microbiome alterations in vertebrates (humans, laboratory fish and mice) in response to diet have been reported earlier (Bolnick et al., 2014). We observe higher *Bacteroidota* abundance post-INU treatment in females, which is consistent with previous findings (Shastri et al., 2015). These selective effects might also be ascribed to differences in the availability of peptidyl-nitrogen in the distal gut of males and females, which in turn act as a source of ammonia to selectively stimulate certain bacterial taxa *viz.*, *Prevotella* and *Barnesiella* within *Bacteroidota* phylum (Kalmokoff et al., 2015; Shastri et al., 2015). Nonetheless, within the same phylum, we also observe an abundance of genus *Bacteroides* as a function of the diet without any influence of sex. Inulin-induced increase in *Bacteroides* is closely associated with its ingenious ability to catabolize complex polysaccharides (Du et al., 2020; Shao et al., 2020). Likewise, increased *Parasutterella* and *Dubosiella* abundance after inulin intake with their direct association with SCFAs levels has been reported before (Li et al., 2020). Conversely, in line with this study, sex dependency of *Turicibacter* and *Enterococcus* genera in C57BJ/6 mice had been documented (Elderman et al., 2018). Although *Faecalibaculum* and *Lactococcus* abundance is decreased more markedly in females, both taxa have been reported to decline in both sexes of mice fed on high-amylose wheat diet (Lim et al., 2021). However, such sex-specific effects are strikingly evident at high fiber doses, suggesting dose-dependency as another variable for exerting universal benefits. Moreover, inulin mediated sex-specific impacts gut microbiome is not only confined in males but also reported in other experimental animal models like adult Wister rats (Massot-Cladera et al., 2020) and *Drosophila melanogaster* (Dong et al., 2019). Besides, inulin supplementation to neonatal piglets had also shown sexual dimorphism in immune development, with females exhibiting higher expression of E-cadherin, CD172^+^ and CD4^+^ antigen-presenting cells compared to males in the distal jejunum (Christoforidou et al., 2019). Pronounced restructuring in the microbiome of females compared to males also reflects in the upregulation of key pathways such as anhydromuropeptides recycling and heptose biosynthesis, which are associated with the formation of new peptidoglycan and lipopolysaccharide moieties, respectively. Besides, some pathways exhibit considerable connection with metabolites’ abundance in both sexes such as reduced pyruvate fermentation to butanoate pathway with reduced butyrate, increased L-leucine degradation with reduced leucine, and reduced L-methionine biosynthesis with reduced methionine.

Even though the gut microbiome and metabolome are modulated differently by inulin among sexes in this study, the machine-learning approach predicts prebiotic traits conferred by inulin in western-style HFD fed mice. Inulin suppresses the abundance of diet-induced obesity-linked taxa reported earlier such as *Enterococcus, Streptococcus* (Zhang et al., 2017), *Faecalibaculum* (Brennan and Garrett, 2019) and *Odoribacter* (Li et al., 2020; Shao et al., 2020) while fostering certain benign taxa which are suppressed under high-fat dietary regimens such as *Dubosiella, Bacteroides, Butyricimonas* and *Parasutterella,* which are known to be enhanced by short-chain and long-chain inulin-type fructans (Li et al., 2020) and neoagarotetraose, a marine oligosaccharide (Zhang et al., 2022). *Barnesiella* and *Lachnospiraceae* are also documented to be increased by inulin and xylooligosaccharides incorporation in HFD-fed male mice, respectively (Du et al., 2020). *Phocaeicola*, a strict anaerobe discovered recently in the human gut ecosystem, could be involved in prebiotic metabolisms as it encodes glycosyl hydrolyses for degrading complex plant-derived polysaccharides and producing propionate and succinate (Wang et al., 2021; Borgonovi et al., 2022). Although these studies were conducted only in males, it still infers that inulin promotes beneficial taxa in a sex-dependent manner, warranting the validation of preclinical/clinical studies to understand sex-related differences for precision nutritional targeting. Similar outputs are also observed in metabolite profiles with suppression of obesity-associated harmful metabolites including malonate, total bile acids, isobutyrate, isovalerate, and trimethylamine together with the promotion of beneficial metabolites including propionate, acetate, glutamate, and glycine, suggesting the sex-specific functional consequences of the microbiome.

Reduction in branched-chain fatty acids (e.g., isobutyrate and isovalerate) in males’ INU group implies reduced toxic effects on the gut as protein fermentation favors the promotion of these acids under limited supply of fermentable fibers (Koh et al., 2016). Moreover, higher levels of these acids together with malonate are seen in T1D patients (Del Chierico et al., 2022). Reduction in total bile acids is another positive feature of fiber as a higher bile acid pool is associated with the risk of colorectal cancer (Degirolamo et al., 2011). A decrease in trimethylamine (TMA) indicates a favorable outcome as T2D patients are found to have elevated plasma levels of TMA-derived trimethylamine-N-oxide (TMAO) (Tang et al., 2017). The abundance of pyruvate is in concert with previous findings whereby high-fat feeding impaired energy metabolism by causing the metabolic disturbance in the tricarboxylic acid (TCA) cycle associated with competitive inhibition of glucose conversion into pyruvate and energy, which in turn was ameliorated by long-term supplementation of dietary polysaccharides (Chen et al., 2018). The abundance of acetoin is also altered by inulin, implying the remodeling of host energy metabolisms because acetoin (another intermediate product of the TCA cycle) concentration is also affected by aerobic and anaerobic microbes during intestinal fermentation processes (Peng et al., 2020). Methionine, an essential amino acid, is significantly decreased in males compared to the control. Despite its crucial role in regulating cellular function for normal growth and development, excessive methionine levels as typically found in the western diet have been shown to alter the intestine microbiome unfavorably by promoting gut pathogens and compromising mucosal structural integrity (Miousse et al., 2017). Contrarily, dietary restriction of methionine in males correlated with enhanced abundance of *Bacteroidaceae* and *Verrucoccaceae* (Wallis et al., 2020); respective members namely, *Bacteroides* and *Akkermansia* of these families also show an increasing trend in males in the current study. Differences in gut microbiome shift the SCFAs abundance differently among sexes, with butyrate decreasing significantly in males while acetate and propionate increase insignificantly in females. Differences in gut microbiota-associated SCFAs production in male and female mice have also been reported previously following antibiotic exposure (Gao et al., 2019) and fiber supplementation (Zhang et al., 2020; Hutchinson et al., 2023).

The disparate responses of gut microbiome-metabolome interaction to diet and sex are evident based on correlational networks, and this interaction also affected physiological responses. The positive association of fat/lean mass ratio with *Lachnospiraceae* appears to be strain-dependent as some studies link its positive association with obesity (Kameyama and Itoh, 2014) while some reported otherwise (Companys et al., 2021). The positive and negative association of propionate with colon length and liver weight, respectively, complies with earlier studies whereby propionate repressed hepatic lipogenesis (Weitkunat et al., 2016) and ameliorated dextran-sulfate-induced colitis (Tong et al., 2016). An increase in lactic-acid-producing genus *Streptococcus* after high-fat feeding is well documented (Liu et al., 2018), which explains its direct association with lactate as observed in our study. Besides, microbiome-metabolome interplay exhibits considerable modulation before and after dietary intervention for males and females. For instance, the relationship of *Anaerostipes* with butyrate and TBA in males pre- and post-inulin regimen indicates inulin-mediated modulation in microbiome and metabolomic pools. Amongst all, alterations in amino acid metabolism exhibit the widest arrays of correlations with the microbiome. Notably, fecal branched-chain amino acids (BCAAs) such as leucine, isoleucine, and valine closely associate with different taxa including *Desulfovibrio, Ruminococcus, Faecalibaculum, Adlercreutzia,* and *Akkermansia*. Cumulative effects of gut bacterial metabolic activity resulted in a net increase in the abundance of BCAAs in the CTL group, which aligns well with earlier studies reporting increased fecal BCAAs by HFD-feeding (Xu et al., 2020). Circulating levels of BCAAs are reported to increase in T2D cases wherein insulin resistance is promoted via activation of the leucine-induced mTORC1 pathway (Lynch and Adams, 2014). The promotion of glycine in both sexes and its association with *Barnesiella* and *Bacteroides* in INU-F group also hints beneficial effect on the host as its supplementation attenuated insulin resistance and oxidative stress in sucrose-fed rats (El-Hafidi et al., 2018).

In summary, our study corroborates how western-style diet induces sex-specific changes in the gut microbiome-metabolome arrays which appears to correlate with impaired gut and metabolic health. Incorporation of inulin, however, ameliorates these fingerprints by fostering beneficial bacterial taxa together with metabolites which are linked with a healthier metabolic state and function of the host. Importantly, inulin-mediated prebiotic effects in microbiome-metabolome arrays differ significantly between sexes, further emphasizing the need to consider sexual dimorphism in future diet-microbiome studies—which has otherwise been often ignored to date—to validate and comprehend the health effects of functional foods as well as for the development of personalized nutrition strategies.

## Data Availability

All datasets generated for this study are included in the manuscript/Supplementary Materials. All the raw sequencing datasets have been submitted to the NCBI Sequence Read Archive (SRA) public repository database under SRA BioProject number PRJNA902407.
