# Supplementary figures and images for: Dietary fiber modulates gut microbiome and metabolome in a host sex-specific manner in a murine model of aging
Source: Front Mol Biosci. 2023 Jun 15;10:1182643. doi: 10.3389/fmolb.2023.1182643 (PMC10345844; doi:10.3389/fmolb.2023.1182643)

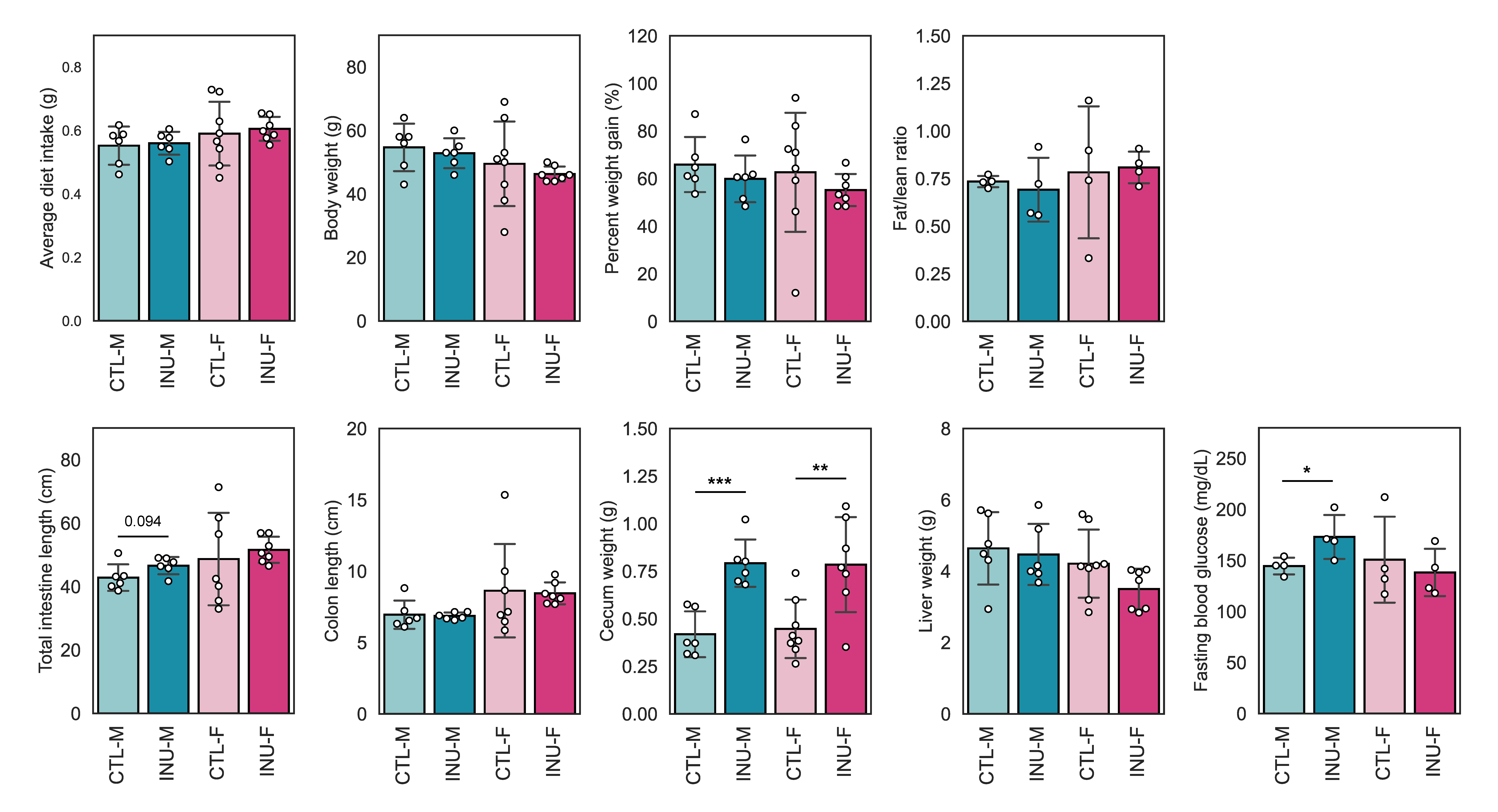

Supplement: Supplementary file 1 [file Image1.TIFF]
